# Dual career experiences of elite coaches enrolled at university level

**DOI:** 10.1371/journal.pone.0283009

**Published:** 2023-04-13

**Authors:** Andrea Fusco, Laura Capranica, Federico Palumbo, Giulio Mosci, Simone Ciaccioni, Mojca Doupona, Cristina Cortis, Flavia Guidotti

**Affiliations:** 1 Department of Human Sciences, Society and Health, University of Cassino and Lazio Meridionale, Cassino, FR, Italy; 2 Department of Movement, Human and Health Sciences, University of Rome “Foro Italico”, Rome, RM, Italy; 3 European Athlete as Student (EAS) Network, Ghaxaq, GXQ, Malta; 4 Department of Sport Sociology, Faculty of Sport, University of Ljubljana, Ljubljana, Slovenia; Università Telematica degli Studi IUL, ITALY

## Abstract

The lifelong education of coaches is one of the priorities of the European Union. Therefore, the purpose of this study was to investigate Italian elite coaches’ motivation to engage in a sport-related academic education, and its demands, barriers, support, and relocation issues in relation to their dual career (DC) path. Sixteen Italian elite coaches (e.g., certified fourth-level national team coaches, senior team coaches) enrolled in a specifically tailored Bachelor’s degree in sports sciences at the University of Rome Foro Italico (Italy) volunteered for this study. A qualitative approach integrating inductive and deductive reasoning, and thematic analysis was applied to participants’ responses to an open-ended item survey. Independently from relocation, student-coaches’ DC perceptions resulted in 15 lower-order themes further organized in 5 high-er-order themes (e.g., Benefit, Challenge, Expectation, Organization, and Support), each related to the contexts (e.g., Personal, Academic, Sport), the DC dimension (e.g., micro, meso, macro, and policy), and the DC push (e.g., facilitating) /pull (e.g., hindering) factors. The elite coaches’ insights emphasize the complexity of the coach lifelong education at university level, and provide valuable information for promoting European and National (e.g., Italian) DC recommendations for elite sportspersons through a cooperation between sport bodies and higher educational institutions.

## Introduction

Over the last decades, sport had the power of shaping and changing the surrounding society, with sports professionals playing an increasingly relevant role in impacting the life of European citizens [1–5]. In fact, physical education teachers and coaches hold an important responsibility in introducing and sustaining active lifestyles of individuals, in promoting values through sport (e.g., gender equality, social inclusion, dignity, freedom, respect for human rights), in supporting talent development, and in preventing drop out and burn out in sports [6, 7]. In this framework, the implementation of educational path-ways to increase the number of qualified sports professionals has become one of the priorities to foster a sustainable development of sport in Europe [6, 8, 9]. In particular, to meet the challenges of this work sector [10], the European Union has recommended quality education, training, and lifelong learning for sports coaches to guarantee the appropriate development of the necessary skills and competences through specific trans-disciplinary knowledge. Thus, in respecting the full competence of the Member States regarding sport policy implementation and the principle of autonomy of sport governing bodies [1, 11], the European Commission recommends including sport within the National Qualification Frameworks (NQFs), in line with the European Qualification Framework (EQF) and international standards [8].

Over the last decades, the education of coaches increasingly attracted sports pedagogy and applied sport sciences scholars interested in defining the learning needs of coaches and the implementation of their education programs [12–17]. Furthermore, structured and extensive dialogue and cooperation between sport bodies and academic institutions is recommended to meet the educational needs and challenges of elite sport coaches by means of: i) fostering the development of skills and competences based educational and/or vocational programs; ii) implementing the application of a wide-range of teaching and/or learning methodologies; iii) harmonizing and recognizing sport educational paths through a variety of learning outcomes encompassing formal learning provided by sport science academic institutions, non-formal learning provided by sports organizations, and informal learning acquired through coaching experience; and iv) promoting mutual sharing of best practices at sports and academic levels [6, 7, 10, 13, 18–20].

On a continuum from novice to expert levels, national sports federations deliver non-formal education and training for coaches, who mainly undergo a professional recruitment based on their previous sports success and/or personal networking, independently from an actual university education in sports sciences [9, 18]. To note, top-level coaches perceive a sport-related academic degree acquired in the early stages of their career as a solid foundation for building sporting success [18]. In considering the European goals of improving the cultural and professional development of sports staff, coaching education is urged to consider a lifelong education through a cooperation between sports bodies and academic institutions [7, 21]. Actually, coaches’ involvement and adherence in lifelong educational paths depend on individual (e.g., interests, needs, motivations, capabilities, efforts, and expectations) and external (e.g., sport cultures and contexts, structure of educational programs, and socio-economic rewards for educated coached) factors [22]. Whilst sports bodies organize educational courses for coaches involving short-term mobility during off-season breaks, formal education at university level is organized over fall, spring, and summer terms of several academic years [23–32]. Thus, the combination of sport and academic careers (e.g., dual career, DC) might be very challenging for elite coaches, especially when the demands of the sport are intensified with increasing competitive levels [23]. In fact, the high mobility characterizing high-level sport and family responsibilities pose additional challenges on elite coaches wishing to enrol at university level [27, 33–44]. Furthermore, despite push (e.g., facilitating) and pull (e.g., hindering) factors influencing DC pathways have been investigated in student-athletes [40, 45], there is lack of research regarding factors facilitating and/or constraining elite coaches’ DC enrolment in formal university education.

Elite sporting success requires countries to adopt a strategic policy to identify, develop, and implement several critical factors, including coaches’ provision and their education [14–46]. Regarding the Italian context, the Country occupies the fifth position in the general Olympic ranking [47], with consistent success in several sports. In this framework, the education of Italian coaches is considered a fundamental element contributing to the national sporting success. Indeed, Italian sport governing bodies deliver sport-specific coach education programs based on the National Qualification Framework (e.g., SNaQ), encompassing defined curricula, assessments, and validation mechanisms according to levels of coaching competence and responsibility in relation to target athletic populations (e.g., youth, sub-elite, elite athletes) [48]. In particular, qualifications of coaches are based on a four-level progression, with defined admission requisites and the acquisition of a minimum of credits for lectures and practical activities for each level, in compliance with the European Credit Transfer and Accumulation System (e.g., ECTS). Thus, each qualification level comprehends sport-specific professional knowledge and defined skills and competences to be acquired in an educational continuum from one level to the other. Specifically, level-1 qualification corresponds to basic training (e.g., assistant coach, mini-mum 10 ECTS), level-2 qualification corresponds to advanced training (e.g., coach, mini-mum 20 ECTS), level-3 qualification corresponds to high level of training (e.g., head coach, minimum 20 ECTS); and level-4 qualification corresponds to national and international coaches (e.g., elite sport coach, minimum 50 ECTS), respectively. In this framework, National Sport Federations (NSFs) deliver sport-specific educational courses for the progression from the first to the third qualification levels, and require certified coaches to attend annual refreshing courses to maintain their qualification. In considering that typically top-level coaching requires a full-time commitment for planning, implementing, and monitoring training and competitions, and extensive interpersonal contact [7, 14, 18], NSFs organize educational courses according to the breaks of their national championships, which could follow different seasonal schedules. To enhance the transfer of knowledge between elite coaches of different sports disciplines presenting outstanding coaching curricula and holding a third level coaching certification [14], the Italian National Olympic Committee (CONI) centrally organizes the fourth-level qualification course, which encompasses seven 1-week modules delivered during a 9-month period to facilitate the coaches’ attendance. Despite in Italy no formal university degree in sport sciences is re-quired to operate in the coaching profession, certified fourth-level coaches are recommended to complement their education enrolling in sport-related academic courses.

Whilst factors positively influencing the engagement of fourth-level coaches at tertiary education could encompass learning opportunities to improve their critical thinking, competencies and skills (e.g., push factors), the lack of DC policies and provisions, and academic constraints requiring full attendance during the fall and spring semesters every year challenge their DC path (e.g., pull factors) [40]. Actually, in absence of formal DC policies at higher education level some Italian universities adopt internal rules and ad hoc DC programs only for elite athletes [49, 50]. To bridge the gap between formal and non-formal/informal coaching education, and to build a sustainable academic career parallel to a high-level coaching career, recently the University of Rome Foro Italico in cooperation with CONI tailored a Bachelor’s degree course (e.g., three years, 180 ECTS) for certified fourth-level sport coaches. In particular, this academic path builds on the acquired high professional knowledge and coaching expertise to achieve a holistic perspective, put-ting emphasis on biomedical (e.g., biochemistry, biomechanics, exercise physiology, functional anatomy, health, and sports medicine), legal-economic (e.g., sports economy and sports law), psycho-social (e.g., neurosciences, sport psychology, and sport sociology), and other sports-related topics (e.g., Olympic sports values, research principles, sports ethics, and sport technology) [51]. Furthermore, to foster authentic learning opportunities for the coaches, learner-centred approaches promoting the transfer of theoretical knowledge into coaching practice are adopted. In compliance with the European guidelines on DC and the recognition of the quest for lifelong education of coaches [6, 23, 52, 53], a blended teaching approach has been deemed appropriate to facilitate the DC path of elite coaches, with on-site classroom sessions (e.g., three 2-week intensive periods during the fall and spring terms), distance learning, a DC tutor, and a general flexibility for attendance and examinations in case of concomitant athletic commitments (e.g., international competitions). Furthermore, a more individualized learning plan is adopted, if needed.

A crucial challenge for DC is to develop policies, programs, and environments at local, national, and European levels to maximize opportunities for sportspersons to pro-mote their personal empowerment through lifelong education [6, 8, 23]. This task requires the involvement of individuals, their entourage, organizations, and communities. To ad-dress the multi-dimensional and complex nature of DC of elite coaches as students, the present study was framed by the social ecological theory that considers the relationship between the individuals and their social, institutional, and cultural contexts [54], and drew upon an articulation of micro- (e.g., the individual), meso- (e.g., his/her interpersonal relationships), macro- (e.g., the sport and education environments), and policy- (e.g., organizational and Governmental policies) dimensions shaping the European DC [25, 26]. Through the social ecological theory lenses, attention to multiple factors at the physical environment (e.g., distance for relocation), the social environment (e.g., culture, economics, and organizational aspects of sports disciplines), and the individual level (e.g., psycho-logical dispositions, behavioural patterns, individuals-entourage relations acting at different levels, interdependence and feedbacks) affecting the DC paths of fourth-level coaches was given. In particular, to uncover the factors positively influencing the engagement of high-level coaches at tertiary education (e.g., push factors) and the challenging factors that might obstacle their DC path (e.g., pull factors) [40], the EU Guidelines on Dual Careers of Athletes, the European Guidelines Regarding the Minimum Requirements in Skills and Competences for Coaches, and the European Sport Coaching Framework have been considered in light of a critical realistic approach, which recognizes multiple realities and lifelong education as a socio-personal process [6, 7, 23, 55, 56]. In this context, a qualitative methodology could be suitable to have a deep understanding of the DC path of elite coaches [18, 44, 56, 57].

Specifically, the aim of this study was to explore Italian elite coaches’ experiences of DC in a sport science academic path. The main research questions were related to the elite coaches’ motivations and benefits to engage in a sport related academic path, and on their perceived demands, barriers, and support in relation to their DC. Furthermore, in extending the aims of the EU-funded collaborative partnership specifically addressing the evidence and eminence on the push/pull factors of “Athletic Migration: Dual Career and qualification in sport (AMiD; 590400-EPP-1-2017-1-AT-SPO-SCP)”, the views and needs of elite coaches as students in relation to temporary relocation were investigated. In particular, elite coaches’ perceptions of their DC experiences could provide valuable insights for promoting the adoption of European and National (e.g., Italian) recommendations on lifelong education of coaches in tertiary education and to enhance the general DC culture in sports, potentially stimulating sport bodies and higher educational institutions to offer effective support to student-coaches [6].

## Methods

### Qualitative approach to the problem and methodology

For the present study, a cross-sectional qualitative approach was applied to investigate DC experiences of elite Italian DC coaches. In particular, a criterion-based sampling was adopted to produce a homogenous sample who was experiencing a DC path and/or a relocating phenomenon, considering the following inclusion criteria: i) being an elite Italian fourth-level coach; and ii) being a DC student-coach enrolled in the sport sciences Bachelor’s degree offered at the University of Rome Foro Italico, specifically tailored for Italian fourth-level coaches.

Qualitative analysis has been applied to develop a contextualized understanding of the DC of student-coaches, including the decision to engage in a DC in relation to their current sport career stage, the preparation and management of their academic and sport demands, the necessary DC support, and the perceived evaluation of their DC path. In this framework, the present study intended to integrate and expand previous research findings regarding the DC of student-athletes to student-coaches [23–32], also in relation to DC push/pull factors [40].

In considering the relevant time demands and efforts elite student-coaches have to face in managing their DC, the application of an open-ended survey was deemed the best option for data collection. In fact, according to the literature [58] open-ended questions do not constrain respondents with specific answer choices, but rather an empty text entry field offers the opportunity to freely express perceptions and opinions. Furthermore, this tool provides a flexible format, giving respondents the chance to reflect before formulating an answer, without time constrains and/or adding meetings to their dense academic and coaching agenda. Therefore, an open-ended survey was considered the most convenient and valuable tool for eliciting participants’ perceptions and opinions in relation to their DC experiences, the push-pull factors, issues, challenges, problems, concerns and needs. Finally, this approach enabled researchers to consider different insights and experiences of single coaches as students and to define shared attributes between them [18, 56].

To ensure the qualitative excellence and the scientific rigor in the present study [59, 60], ethical standards have been guaranteed by the external approvals of the European Commission selecting the AMiD project for financing, and the Institutional Review Board of the Department of Human Sciences, Society, and Health of the University of Cassino and Lazio Meridionale (approval number: 243.3R; date of approval: 01.15.2020). Furthermore, the open discussion between the members of the research team (e.g., very experienced scholars on DC research and on coaches’ education) was considered crucial to clearly outline the research context and questions, to develop the open-ended survey (e.g., proposing questions, reaching a consensus on the clarity of the content and clarity of the wording), to purposefully recruit the participants, and to define clear procedures for data extraction and analysis with no pre-conceptions and/or interreferences with participants’ opinions. According to the social-ecological theory [54], questions pertained the physical environment (e.g., distance for relocation, accommodation), the social environment (e.g., culture, economics, and organizational aspects of DC), and the coach’s motivations, behaviours, and personal relations with family members, teachers, and athletes.

### Participants

Sixteen Italian elite fourth-level coaches (women = 2, men = 14; age 49.0 ± 9.2 yrs.) enrolled in the sport sciences Bachelor’s degree offered at the University of Rome Foro Italico (Italy) specifically tailored for Italian elite coaches volunteered for the study. Participants reported a coaching experience of 20.7 ± 10.4 yrs. at eight NSFs (Table 1). Whilst four coaches held university degrees in areas not related to sports (e.g., philosophy, law, medicine with specialization in cardiology, political and social sciences, and communication sciences), the majority of the participants (e.g., 75%) declared to have a secondary education level. Furthermore, six coaches were leaving in the town of the University, whereas the remaining 10 relocated from a distance of 420 ± 192 km.

**Table 1:** Characteristics of the participants, their educational level, and distance between hometown and the university town.

Sex	Age	Sport	Highest Academic Degree	Distance (km)
M	63	Rowing	High School	315
M	54	Karate	High School	550
W	49	Canoeing-Kayak	University	693
M	39	Kickboxing	High School	359
M	33	Modern Pentathlon	High School	0
M	42	Fencing	High School	0
M	50	Track & Field	University	0
W	37	Judo	High School	315
M	54	Track & Field	High School	316
M	59	Chinese martial arts	University	280
M	46	Rowing	2-University	0
M	54	Tennis	High School	256
M	52	Badminton	High School	801
M	64	Tennis	High School	315
M	39	Canoeing-Kayak	High School	0
M	49	Beach Volley	High School	0

M: men; W: women

### Procedures and data generation

To meet the principles of connectivity, humanness and empathy favouring the interaction between the interviewer and the interviewed [61], a member of the research team having experience in the selected qualitative methodology, working as a high-level coach at national and international level for the past 15 years, holding an academic degree in sports sciences acquired as a student-coach, and having previous acquaintance with the participants was deemed appropriate to introduce the survey to the elite coaches in an on-line modality. Participants were provided a 5–10 min presentation regarding the aim of the study and their expected contribution, highlighting the relevance of their perceptions and opinions to shed a light on DC paths of elite coaches as students. Participants were ensured that participation was voluntary, the confidentiality of their responses, the right to refuse to answer specific questions, and the option of dropping out at any time without providing any reason. After having provided a written informed consent, each participating coach reported information on previous career experiences to get a general background. Hence, five main questions conceptualized on previous findings on student-athletes’ DC and coaches development pathways [18, 39, 40] were posed in the open-ended survey:

1. “Why did you decide to become a student-coach?”;
2. “How did you prepare for your DC path?”;
3. “What kind of support did you receive for your DC?”;
4. “What went good and what went bad during your DC path?”;
5. “Do you have recommendations/messages for a future DC coach-athletes to have a positive DC experience?”.

These questions were intended to elicit information regarding the coach’s perceived push and pull factors related to their DC physical and social environments, and their in-dividual motivation, goals, expectations, actual challenges at professional and logistic levels, and support received at the personal (e.g., family, peers, friends), academic (e.g., DC tutor, teaching staff) and sport (e.g., sport club, NSF, Olympic Committee) levels. Finally, participants were given the opportunity to provide any additional information they felt was necessary, had been missed, or posited any inquiries they had about the topic, the survey, and/or the data collection process. To avoid potential bias in the analytical pro-cess, two members of the research team engaged in repeated, recursive readings of all survey transcripts to reach a consensus on the identification and revision of the major themes.

### Data analysis

A preliminary data extraction was conducted to identify the source of information for the participants on the academic path for student-coaches and the motivation for enrol-ling in the university program, as well as the challenges encountered by relocating coaches. According to the literature [62], inductive (e.g., coaches’ personal experiences) and deductive (e.g., conceptual framework) data analyses were used, with the thematic analysis encompassing six phases: familiarization, coding, theme development, refinement, and naming, and writing up [56]. Then, individual raw quotes were coded and categorized into sub-themes, which were further aligned deductively with the conceptual lens (e.g., motivation, challenges or barriers, support, decision-making). To prevent potential bias in the analytical process, strategies to minimize researcher bias included multiple readings of the text by two authors, and the extraction of major themes that were discussed until consensus was reached [62]. Whilst a researcher facilitated the credibility of data analysis through being an elite coach who experienced DC as well as relocation and migration for coaching purposes, the other engaged as a critical partner and encouraged reflexivity by challenging each construction [60].

## Results

Participants in the study represented mainly individual sports disciplines (n = 13; 81%), whereas three coaches only were involved in team and/or mixed individual-team sports. In general, the elite coaches declared that official communication from the Olympic Committee/NSFs provided information on the opportunity to enrol in a sport-related academic path with DC services (n = 12), followed by word-of-mouth information from other coaches (n = 3), and internet (n = 1).

Regarding the motivation to enrol in a DC program (e.g., push factor; Table 2), results showed that 56% of the respondents were pushed by the opportunity to improve the level of knowledge, competencies, and skills to ameliorate their coaching preparation. Furthermore, 25% of the coaches considered personal development and satisfaction as the most important reasons to enrol in a university degree. Conversely, 19% of the sample indicated that acquiring a university degree would have fostered their professional career progression and recognition. Finally, data showed that younger coaches deemed more relevant education to ameliorate their competences and skills with respect to their older counterparts.

**Table 2:** Motivations (e.g., push factors) of student-coaches to enrol in a sport-related DC academic path in relation to the age and sport discipline.

Respondents	Sport typology	Motivation to be a student-coach
n = 9 respondents Age 46.8±9.7	Individual	To achieve the maximum level of competences
	Individual	To constantly ameliorate my preparation
	Individual	To achieve the maximum level of competences
	Individual	To have a higher education degree in sports sciences to be a good coach
	Individual	To complete and give a better structure to the education of high-level coaches
	Individual	To consolidate my knowledge in sport, despite I have two university degrees
	Individual	To ameliorate my knowledge
	Individual	To ameliorate knowledge not related to my discipline
	Individual	To complete my knowledge and skills with a university degree in sport sciences
n = 4 respondents Age 52.8±5.4	Individual	Personal satisfaction and full development
	Individual	Personal development
	Individual	To complete my personal development
	Mixed	Personal satisfaction
n = 3 respondents Age 50.7±12.6	Mixed	To ameliorate my professional position at the sport club
	Individual	To have a higher education degree in sports sciences for my coaching position
	Team	Optimal opportunity for a professional development in sport

Independently from relocation, data regarding student-coaches’ perceptions concerning their DC path resulted in 15 lower-order themes, further organized in 5 high-er-order themes (e.g., Benefit, Challenge, Expectation, Organization, and Support), in relation to the context (e.g., Personal, Academic, Sport), the micro, meso, macro, and policy DC dimensions, and push/pull factor identification (Table 3).

**Table 3:** Lower- and higher-order themes (e.g., benefit, challenge, expectation, organization, and support), organized in relation to the contexts (e.g., personal, academic, and sport), and the dual career dimensions (e.g., micro, meso, macro, and policy).

Lower-order	Higher-order	Context	Dimension	Push/Pull Factor
Higher education degree in sport sciences	Benefit	Personal	Micro	Push
Difficulty in committing to study	Challenge	Personal	Micro	Pull
Finance and logistics	Challenge	Personal	Micro	Pull
Lack of family life	Challenge	Personal	Micro	Pull
Lack of social life	Challenge	Personal	Micro	Pull
Support from family	Support	Personal	Meso	Push/Pull
Support from professors in a DC path	Support	Personal	Meso	Push/Pull
Transfer of knowledge between elite coaches	Benefit	Sport	Meso	Push
Arrangements with a substitute coach	Challenge	Sport	Meso	Pull
Difficulty in combining sport and academics	Challenge	Sport	Macro	Pull
Difficulty in organizing training sessions	Challenge	Sport	Macro	Pull
Professional upgrade upon graduation	Expectation	Sport	Policy	Push
Online educational material	Organization	Academic	Macro	Push/Pull
Flexibility for exams	Organization	Academic	Macro	Push/Pull
Academic provision and services	Support	Academic	Macro	Push/Pull

At personal level, six lower-order themes encompassing three higher-order themes (e.g., benefit, challenge, and support) emerged, five pertaining the student-coaches themselves (e.g., micro dimension), and one pertaining the support received from their families (e.g., meso dimension). Relocating student-coaches reported issues related to the costs of travelling and accommodation (e.g., pull factor), with logistic and financial challenges reflected in the next quotes:

*“To find a good accommodation close to the university was challenging”.*

*“The possibility to sustain travel expenses is crucial*, *especially in considering that during the on-site periods earned income is lacking”.*

*“Special arrangements to accommodate relocating athletes and coaches are necessary for this type of academic courses”.*

*“The class schedule should be arranged to reduce and facilitate the transfer of relocating student-coaches”.*

To overcome partially these challenges, the participants declared to have adopted the solution of group booking, which helped them reducing the costs of accommodation.

Independently from relocation, the financial burden to engage in a long-term academic path emerged (e.g., pull factor):

*“It is often difficult to combine work and education and the choice is directed obviously on the side providing an income”.*

Furthermore, in starting an academic path with advancing age, student-coaches were challenged in their capability to commit in studying for a long time, and had a very limited family and social life (e.g., pull factors):

*“With advancing age*, *your capability to study many hours in a row is reduced”.*

*“I feel a lack of time for my family”.*

*“I encountered some problems in organizing training sessions for my athletes and lack of time for my family”.*

Despite the challenges, the student-coaches really valued their academic engagement (e.g., push factor), especially when in the past the participant had dropped-out the university due to a sport career:

*“As a teenager*, *I had to interrupt my studies due to my sporting commitments (study burn-out*)”.

On the other hand, the participants declared to have received support from their family, which presence was considered determinant to sustain DC efforts (e.g., push/pull factor):

*“My family supported my path”.*

*“My wife’s encouragement made a difference in my decision to undertake this wonderful course”.*

At sport level, five lower-order themes encompassing three higher-order themes (e.g., benefit, challenges, and expectation) were found, four pertaining the relationships of coaches with their academic and professional peers (e.g., meso dimension), two pertaining the difficulty to manage sport and DC commitments (e.g., macro dimension), and one pertaining the expectations from the sport bodies (e.g., policy dimension). Student-coaches appreciated the opportunity to share experiences and visions with their peers from other disciplines (e.g., push factor):

*“I appreciated the great relationship with colleagues […]”.*

*“Exchanges and comparisons between sport disciplines should be expanded because they allow an exponential growth”.*

*“More time should be devoted to methodological exchanges between coaches”.*

*“In relating with high-level coaches of other sports I have been acquainted with new training methodologies”.*

*“[…] also because being able to interact with other high-level coaches is definitely an added value of the course”.*

*“On a personal level*, *having been in contact with many people who loved their sport enriched me”.*

*“I learned a lot from my academic study and from my classmates”.*

In addition to DC challenges, coaches experienced difficulties in managing their coaching schedule (e.g., pull factor), especially when they had to plan, implement, and monitor training sessions. In particular, four participants declared that they hired a coach to administer and supervise the training sessions of their athletes, which might have affected the quality of training and/or have caused inter-personal problems with athletes:

*“I faced difficulties in organizing university commitments to the detriment of the sports ones”.*

*“Neglecting my work was really the very negative aspect of this academic experience”.*

*“Problems related to the teams I coached in my hometown”.*

*“I faced challenges to organize training sessions”.*

*“I feel that my sport career could have been jeopardized”.*

Nonetheless, the student-coaches appreciated the positive effects of attending a sport-related university path for improving their technical and pedagogical capabilities, and they had expectations for future improvements of their job positions (e.g., push factors):

*“The course allowed me to evolve as a sport coach and as a person*”

*"This course was a stimulus to face new challenges and to share different experiences*. *In my opinion*, *sharing experiences and knowledge between expert coaches of various sport disciplines should be expanded”.*

*“[…] There should be the possibility of being inserted in a working context for which I have studied*. *In being even more qualified*, *I expect that sport bodies can profit from this greater competence acquisition”.*

At the academic level, four lower-order themes encompassing three higher-order themes (e.g., organization and support) were found, two pertaining the availability of online study material and flexibility for exams (e.g., macro dimension), and two pertaining the inter-personal support (e.g., meso dimension) and logistic support (e.g., policy dimension). Student-coaches appreciated the support they received from professors and classmates, the arrangement of distance learning, and the flexibility for examinations, which presence was deemed determinant for their DC (e.g., push/pull factors). However, some logistic and financial support were envisaged (e.g., push/pull factor).

*“I have found excellent professors and some very relevant topics even though some of them were less interesting*, *A better cooperation and coordination between university*, *sport federations and sport clubs is advisable*. *Institutional relationship*, *if not some university scholarship for elite coaches*, *should be available”.*

*"Education of coaches is crucial even though it requires relocation”.*

*"Very positive aspects encompassed the acquaintance of other high-level coaches*, *with a consequent increase in motivation to work in an even more scientific way”.*

*“I appreciated the great relationship created with my colleagues and the great availability of the professors”.*

*“This academic path increased my motivation to study training patterns”.*

Regarding push and pull factors in relation to the lower-order themes, results reported factors facilitating coaches’ DC (e.g., push factors) accounting for 20% (n = 3), including the perceived value of a sport-related academic education, peer education with other elite coaches, and the potential professional upgrade upon graduation. Conversely, an uncertain role (e.g., either a push or a pull factor) emerged for 33% (n = 5) of the lower-order themes, with family and professors support, and DC academic services representing a determinant in elite coaches’ DC enrollment. Finally, pull factors accounted for 47%(n = 7), with difficulty in studying as middle-aged individuals, additional expenses, reduced social and family life, difficult arrangements in their coaching activity, and efforts in managing academic and sport duties playing a crucial role in constraining coaches’ DC path.

## Discussion

The present findings on Italian elite coaches enrolled in a Bachelor’s degree in sports sciences represent a novel approach to DC and lifelong education in and through sports, extending the recommendations of the EU guidelines for DC of athletes and the EU recommendations for coach education [6, 23]. In fact, the demand for and importance placed upon high-quality education for coaches is increasing, with lifelong learning considered a key pillar for the professional advancement in relation to inter- and intra-personal knowledge development in sport coaching [6, 7, 63, 64]. Despite several studies focused on tertiary education of coaches, there is limited information on the coaches attending these courses, and their expectations and perceptions regarding the push and pull factors for enrolling and progressing towards a university degree [65, 66]. In fact, middle-aged elite coaches might be challenged to combine their university education with full-time commitment in managing top-level athletes and family obligations [7, 14, 18, 23, 52]. Through the social-ecological theory lenses [54], the present findings integrate Italian elite coaches’ efforts to lifelong education at university level with DC environment-focused interventions to enhance their physical and social surroundings. sheading a light on the dynamic inter-play among persons, groups, and their socio-physical milieus. Therefore, in examining the perceptions of Italian high-level coaches enrolled in a higher education program in sports sciences, the present study represents an important contribution to stimulate the conversation on the development of the DC framework [67–69], extending the DC debate to the wider figure of the elite sportsperson. In fact, to enrol in a formal higher education path for developing additional competences to sustain their role in high-level sport [6], elite coaches need specific DC support to manage their professional and academic duties, as well as their family role. Finally, similarities and differences from the outcomes of previous related research on elite coaches and DC athletes could increase the generalizability of results [44, 52].

The analysis of the interviews of the survey showed that elite coaches are very motivated to implement their competences and skills. Furthermore, in considering the availability of on-line degrees in sports sciences, coaches’ choice of a blended format indicates the high perceived value of face-to-face learning conditions, which favours inter-personal relationships between elite coaches and academic staff and peer education. In fact, all participants opted for a three-year degree in presence, overcoming the difficulties of relocation during the on-site periods. Despite their highest formal education was secondary school degree, through a well-structured non-formal and informal sport-specific education organized at sport level (e.g., the Italian NSFs and Olympic Committee), the participants in this study held the highest coaching certification [10, 48] and many have achieved success at national and international levels. Nevertheless, high-level coaches perceived a formal education as an added value to complement their knowledge to nurture their coaching professionalization. In particular, the main motivation for enrolling in a Bachelor’s degree in sports sciences was the expectation to deepen their knowledge on the scientific and methodological aspects relevant to high-level sports for their personal and professional growth. These findings are in line with the literature [6, 7, 18, 70] reporting high motivation for self-improvement and permanent on-going education as a primary reason of coaches to enrol in an academic path, making professional development inseparable from the pro-cess of increasing coaching competency through tertiary education [18].

To pursue sport success coaches usually work and/or collaborate with many experts (e.g., other coaches, physical trainers, sport staff, medical staff, managers), requiring adequate competences to build effective interpersonal communication based on common knowledge and technical languages [63]. Despite elite coaches familiarize with many knowledge areas during their non-formal and informal coaching education and experience, participants reported that a formal academic path is a valuable opportunity to further deepen their understanding of specific aspects relevant to high performance sports. Furthermore, according to the literature on adult students learning [71], coaches in this study highlighted difficulties in engaging in an academic path after many years from the end of their previous high school or university experience, perceiving a decrease in their capability to understand and retain the course contents. Actually, multi-disciplinary educational programs encompassing different scientific languages and fostering critical thinking could nurture open-mindedness, although putting additional learning challenges in terms of communication and work processes [72]. Furthermore, the reported positive interaction with classmates and professors determined a constructive learning environment, which allowed a cooperative climate respecting the coach’s needs [73]. Indeed, awareness of the inherent and extrinsic problems faced by coaches helps educators developing their teaching skills and styles, encouraging individual and collective flourishing [71, 74].

Similar to student-athletes [24], the present findings confirm that DC poses a constant tension between the academic, sporting, and social components of the student-coaches’ life. In fact, elite coaches reported challenges particularly related to the financial burden, compelling sports obligations, limited leisure time, and overload feelings [24–32]. As most employees wishing to continue their education as student-workers, elite coaches need to develop their own approach to manage their family, work, and other social responsibilities. This aspect could be exacerbated for female coaches in considering their traditional role in the family care [71, 75]. Actually, the unbalanced gender representation emerged in the present study confirmed the men hegemony pervading the sporting occupational sector, especially in relation to high level sport [76–78], probably due to gender stereotypes affecting female coaches career advancement, and barriers experienced at individual, interpersonal, organizational/structural, and socio-cultural levels [75, 79, 80]. In line with DC literature, the support from family was reported as relevant in helping them envisaging effective strategies to resolve the problems and providing continuous encouragement for accomplishing a DC path [24, 42, 81, 82]. In this respect, there is a need for a full recognition of the complexity and challenges of DC paths from both academic and sport organizations, and to foster the inter-institutional dialogue and cooperation to shape a sustainable DC environment for coaches. Moreover, scholarships allocation and financial support might represent relevant tools to reduce the financial burden of coaches’ tertiary education [6, 23, 68].

Continuing education and migration for career development is an ongoing phenomenon, driven by the increasing demands of elite sports and sport globalization for athletic, financial, and cultural mechanisms [6, 44]. The present study emphasizes the im-portance of harmonizing the coaches’ education within a specific DC context, especially for coaches relocating for sport and/or academic reasons. Indeed, it is challenging to design a standardized academic program for elite coaches without considering their idiosyncratic learning pathways [7, 18, 52, 83]. To organize a DC path for elite coaches it is necessary to consider that sport commitments require time-consuming physical presence for training and competitions according to the sport-specific season planning. Thus, the content, the organization, and the teaching methodologies should be based on a thorough consultation with all relevant stakeholders (e.g., CONI, NSFs, coaches, athletes, university, professors, administrative staff) to provide a blended learning program, allowing inter-personal relationships in the classroom, e-learning provision, exam flexibility, tailored courses’ content based on coaches previously acquired level of knowledge and expertise, and a continuous bi-directional feedback to facilitate eventual necessary adjustments [6, 23].

Despite the encountered challenges, all the coaches reported a positive cultural experience through cross-sport and academic collaborative interactions, which was substantiated by the final achievement of a university degree and a personal and professional growth [84]. Even though this experience was facilitated by interpersonal relationships and support from family and academic staff, the coach’s employment status, the lack of support and recognition from NSFs and sports clubs, the absence of logistic and organizational arrangements, and the economic burden were reported as key destabilizing factors for the academic success, which were resolved by the coach’s own motivation and individual adaptation. In addition to the coach’s personal capability to adapt and find solution for relevant modifications in practices and attitudes, a match between the coaches’ motivation, personal circumstances (e.g., familial status, finances, etc.), and support at personal and academic levels have to be considered.

The present findings could represent a starting point for the implementation of coaches’ education paths, which is often demanded to NSFs. To note, in this study the NSFs did not play any role in raising the awareness of their high elite coaches regarding the opportunity of a DC path, did not support their economic and logistic aspects, and did not recognize merits to those coaches who achieved a Bachelor’s degree in sports sciences. Therefore, it is not surprising that the number of coaches enrolling in a three-year DC university path is limited, obstructing the implementation of European recommendations for coaches’ education. Therefore, NSFs need to take their responsibilities in encouraging a life-long education of their elite coaches, especially if they hold responsibility positions with high-level teams. In general, the present study showed that sport responsibilities challenged all the student-coaches in organizing their sport commitments. Indeed, high performance coaching revolves around the management of the performance team, including other coaches and support staff. Despite student-coaches could delegate momentarily their role, they have to avoid the risk to compromise their position and/or the quality of their coaching plans when not supported due to their DC obligations. Sport organizations should increase their role in facilitating coaches in combining their working commitments with academic education for a general advancement of the sports culture and to nurture the development of qualified sports professionals [6, 9, 85]. In fact, university courses specifically tailored for elite coaches could not only allow a constant interaction between col-leagues of different sports disciplines, but also provide opportunities for envisioning further cooperation and innovative practices [52, 74]. Success stories and role models (e.g., successful DC coaches) should be promoted to boost the establishment of cooperation agreements between academic and sport institutions and to encourage other coaches to pursue a DC path [86]. Furthermore, the student-coaches’ motivation during their DC path has to be monitored to maintain the mental toughness necessary to overcome challenges and barriers. Finally, according to the Transformational Learning Theory supporting coaches in empowering athletes through mentoring, inspirational motivation and intellectual stimulation, student-coaches could be role models for student-athletes in their DC paths at tertiary level, contributing to the advancement of European DC [23, 31, 67–69, 86].

## Conclusions

The present study generated new evidence regarding the sustainability of the DC framework and could foster the debate regarding the extension of DC to the wider figure of the elite sportsperson. In fact, to our knowledge the present findings represent the first at-tempt to investigate DC experiences of elite coaches, complementing existing literature regarding the combination of sports and education in elite student-athletes. Some limitations for the generalizability of the findings should be considered (e.g., the application of an open-ended survey, the limited number of coaches participating the study, the unbalanced gender representation), which could serve as a guidance for future research on DC pathways of elite coaches. Despite the participants were successful coaches at 8 NSFs, the sport typology also might have limited the generalizability of results. Therefore, future studies including a higher number of both individual and team sports are recommended, also considering the socio-economic impact of the sports disciplines within the national context.

To strengthen the evidence on the interaction between coaches at academic level, future studies should consider the stratification of a larger experimental sample and provide further insights regarding the challenges and needs of coaches’ DC path. Furthermore, follow-up studies should evaluate the actual integration of acquired formal knowledge into sport-specific programs elite coaches implement for their athletes. Finally, in considering that coach education and DC policies differ considerably between European countries, future research on the individual, inter-personal, environmental, and policy dimensions affecting constructive DC paths for coaches is strongly needed to move towards the establishment of a culture that supports lifelong education for coaches in light of their relevant role in society [6, 9, 25, 67–68].
